# ARAS: an automated radioactivity aliquoting system for dispensing solutions containing positron-emitting radioisotopes

**DOI:** 10.1186/s13550-016-0176-9

**Published:** 2016-03-09

**Authors:** Alex A. Dooraghi, Lewis Carroll, Jeffrey Collins, R. Michael van Dam, Arion F. Chatziioannou

**Affiliations:** Crump Institute for Molecular Imaging, University of California, Los Angeles (UCLA), Los Angeles, CA 90095 USA; Department of Molecular & Medical Pharmacology, University of California, Los Angeles (UCLA), Los Angeles, CA 90095 USA; Present Address: Lawrence Livermore National Laboratory, Livermore, CA 94550 USA; Carroll and Ramsey Associates, Berkeley, CA 94710 USA

**Keywords:** Radiation detection, Automation, Aliquot, Dispense, Positron, Beta particle, PET, Positron emission tomography

## Abstract

**Background:**

Automated protocols for measuring and dispensing solutions containing radioisotopes are essential not only for providing a safe environment for radiation workers but also to ensure accuracy of dispensed radioactivity and an efficient workflow. For this purpose, we have designed ARAS, an automated radioactivity aliquoting system for dispensing solutions containing positron-emitting radioisotopes with particular focus on fluorine-18 (^18^F).

**Methods:**

The key to the system is the combination of a radiation detector measuring radioactivity concentration, in line with a peristaltic pump dispensing known volumes.

**Results:**

The combined system demonstrates volume variation to be within 5 % for dispensing volumes of 20 μL or greater. When considering volumes of 20 μL or greater, the delivered radioactivity is in agreement with the requested amount as measured independently with a dose calibrator to within 2 % on average.

**Conclusions:**

The integration of the detector and pump in an in-line system leads to a flexible and compact approach that can accurately dispense solutions containing radioactivity concentrations ranging from the high values typical of [^18^F]fluoride directly produced from a cyclotron (~0.1–1 mCi μL^−1^) to the low values typical of batches of [^18^F]fluoride-labeled radiotracers intended for preclinical mouse scans (~1–10 μCi μL^−1^).

## Background

According to the ORAMED (Optimization of RAdiation protection for MEDical staff) study, nearly one in five workers in nuclear medicine is likely to receive more than the legal dose limit for the skin (500 mSv per year) [1]. To better comply with regulations and to enhance the safety of employees, protocols must be developed that minimize radiation exposure. Automated tools for handling radiation provide a promising approach to reduce radiation exposure [2]. Furthermore, well-implemented automated systems reduce human error and, thus, allow for a streamlined workflow. For clinical applications, systems have been developed such as Intego™ (MEDRAD, Warrendale, PA), which dispenses and automatically delivers a prescribed dose of a radiotracer to a patient. With this system in use for the injection step of PET procedures, whole-body and extremity radiation exposures to nuclear medicine workers were significantly reduced by 38 and 94 %, respectively [3]. Customized tools similar to Intego™ but developed for preclinical PET radiotracer synthesis and usage can be implemented to provide corresponding reductions in whole-body and extremity radiation exposures to radiation workers.

In regard to the development and production of radiotracers, a tool that allows for automated aliquoting of user-specified amounts from a batch of [^18^F]fluoride solution will eliminate the need for radiation workers to manually draw radioactivity. Moreover, this automated dispenser can be implemented in any step of the radiotracer development and usage pipeline, including not only aliquoting of [^18^F]fluoride after cyclotron bombardment to support multiple research or production runs but also aliquoting the radiotracer for delivery into a subject. However, a technical challenge faced in both of these applications is the small volume of original stock solutions and the even smaller volume of individual aliquots. For example, [^18^F]fluoride from the cyclotron may be delivered in as little as ~1 mL (or even down to several hundred microliters, depending on the cyclotron target design) and a batch of a PET probe for preclinical imaging in mice may be concentrated in ~1 mL. The volume for mice should typically be no more than 100 μL, and since the batch is potentially used over the span of several half-lives of F-18, this means the initial aliquots will have a significantly smaller volume, down to a few 10s of microliters.

To address the opportunity of significantly increasing safety and accuracy, we have developed ARAS, an automated radioactivity aliquoting system for dispensing solutions containing positron-emitting radioisotopes with particular focus on fluorine-18 (^18^F). ARAS consists of a solid-state radiation detector in series with a peristaltic pump. The detector comprises two 3 × 30 mm^2^ anti-parallel PIN Si diodes operated in current mode. Two diodes are used in order to suppress the background from long-range 511-keV photons produced from positron-electron annihilation. These are present when handling positron-emitting radioisotopes like ^18^F which is commonly used in PET and is the radioisotope considered in this work. For each batch of radioisotope, the detector is used to perform a one-time calibration to determine the initial reference radioactivity concentration. The peristaltic pump is used to deliver prescribed volumes of [^18^F]fluoride solutions based on the decay-corrected radioactivity concentration and the desired amount of radioactivity. The automated design of this system promises to reduce exposure to the operator compared to manual dispensing operations and manual measurements using a dose calibrator. In this work, we describe the design of the prototype system and characterize the system performance. We also present preliminary examples of possible usage in radiochemistry and in mouse tail vein injections.

## Methods

### System design

**Fig. 1:**
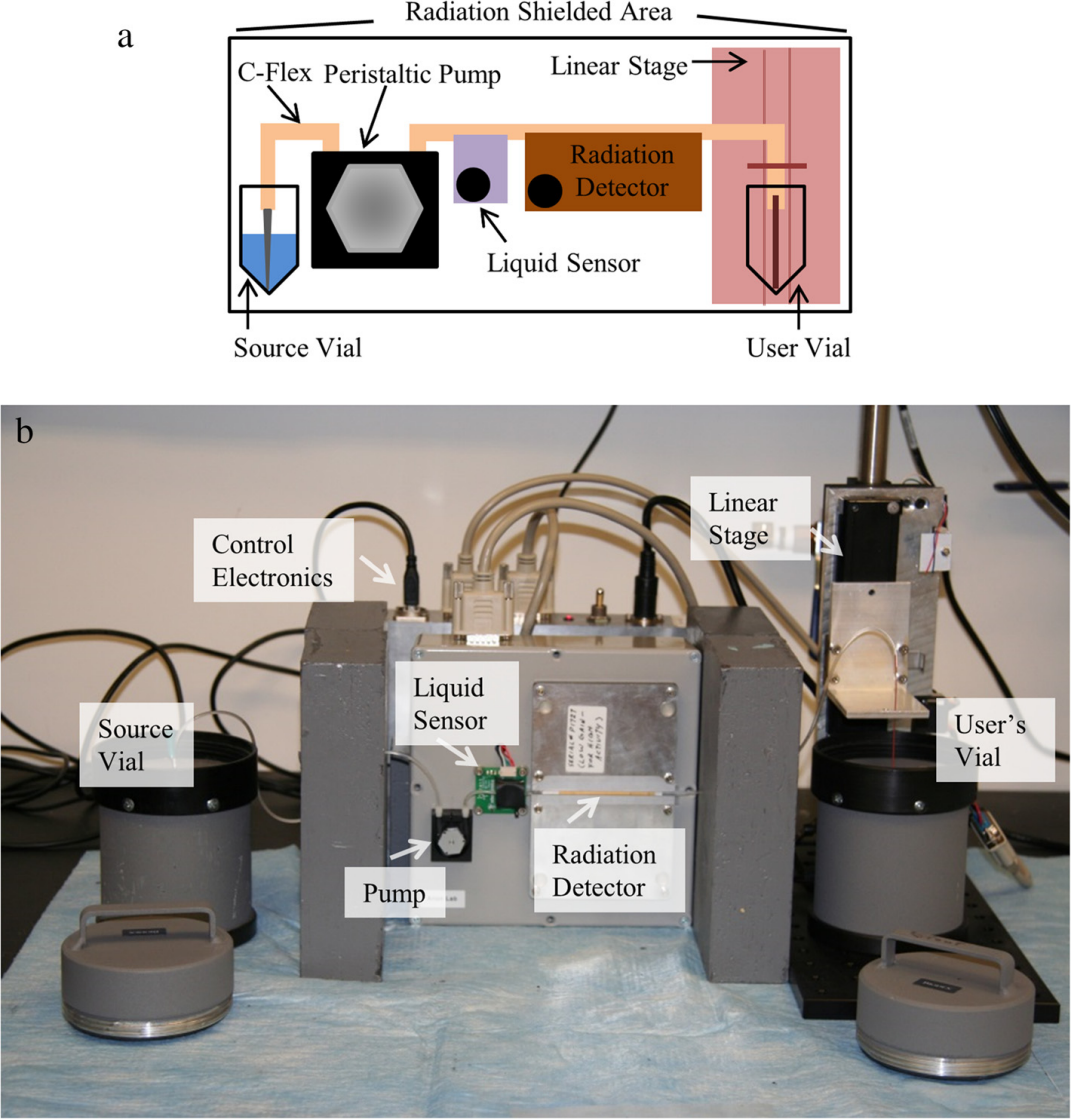
Illustrations of the main components of ARAS. **a** Schematic highlighting the main components of the dispenser system and **b** photograph of the system

ARAS was designed to automate aliquoting of solutions containing positron-emitting radioisotopes such as [^18^F]fluoride. Figure 1a shows a schematic of the key functional components of the dispenser. C-Flex tubing (TS020C, Instech, Plymouth Meeting, PA) was used to connect the input source vial via a needle to the output user vial. At the end of the C-Flex tubing, either polyether ether ketone (PEEK) tubing (1569, IDEX Health & Science, Oak Harbor, WA) or a catheter (0099EO, ReCathCo, Allison Park, PA) was used, depending on the application. The C-Flex tubing passed through a peristaltic pump and liquid sensor and passed over a radiation detector. For radiochemistry usage, the C-Flex tubing was subsequently attached to a linear stage. The pump (P625/900, Instech, Plymouth Meeting, PA) and tubing combination provided set flow rates (μL s^−1^) which allowed for calculation of the time necessary to actuate the pump to fill a known section of tubing with fluid. Once the tubing was filled, the actuation time was set to dispense a requested volume, given the known flow rate. Although distribution by volume specification is useful, often, an amount of radioactivity is requested. To dispense in this fashion, a custom-designed radiation detector (see “Radiation detection technique” section) was used to determine the reference radioactivity concentration (i.e., mCi μL^−1^) during a one-time calibration step per batch of radioactive solution. The reference radioactivity concentration was then decay corrected to the time of the radioactivity request. Dividing the requested radioactivity by the decay-corrected radioactivity concentration yields the volume necessary to dispense to the user vial. A liquid sensor (OCB350L062Z, Optek/TT Electronics, Carrollton, TX) was used to provide a reference position for the location of the start of the moving solution. The volume of tubing from the liquid sensor to the output was accurately known, enabling accurate filling of the entire tubing, which was followed by dispensing of the requested amount. For radioactivity aliquoting in radiochemistry applications, a linear stage (LX20, Misumi, Tokyo, Japan) controlled by a high-torque stepper motor (17Y202D-LW4, Anaheim Automation, Anaheim, CA) was used to improve accuracy and safety when handling small aliquots. Specifically, small droplets may remain suspended at the end of the PEEK tubing. To avoid this, the tip of the tubing was positioned in contact with the inner wall of the collection vial during dispensing. This contact assured the droplet would dislodge from the tubing and rest in the collection vial. After dispensing, the linear stage was activated to lift the dispenser tubing. The pump was then actuated in reverse to withdraw the radioactivity back into a lead-shielded section of tubing, without also withdrawing the delivered radioactivity from the user vial. Withdrawal of the radioactivity at the end of dispensing was done to reduce exposure to the operator when he/she reached to retrieve the vial, or when he/she installed a fresh vial for the next dispensing operation.

Figure 1b shows an implementation of the setup. Lead bricks placed in front of the system (not shown) were used to attenuate the exposed radiation emanating from the tubing. For radiochemistry usage, the entire setup was placed in a lead-shielded cabinet. In order to make the pump, optical sensor, and radiation detector footprint as compact as possible, these components were housed in an enclosure separate from power and computer control connections, which could be placed outside of the radiation shielding. A USB DAQ (NI USB-6215, National Instruments, Austin, TX) was used to interface with the peristaltic pump, radiation detector, and optical sensor. An RS-485 to USB converter provided a USB connection to interface with the stage controller. Control software was developed in LabVIEW following an event-driven machine state design pattern.

### Radiation detection technique

**Fig. 2:**
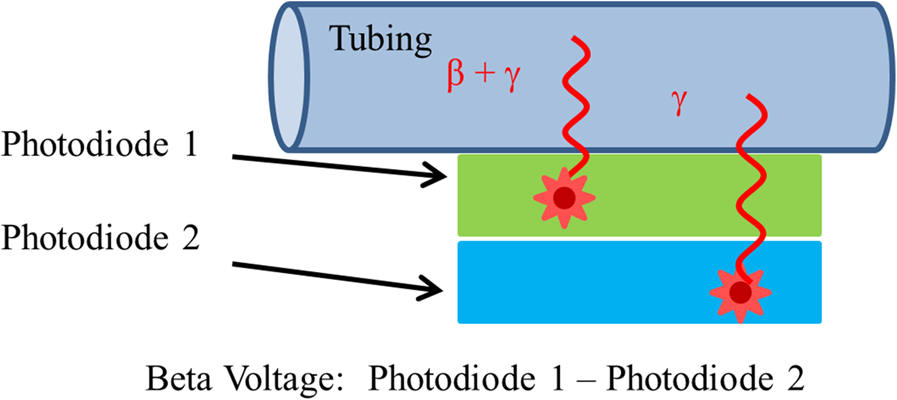
Diagram of the dual photodiode detection method. Photodiode 1 responds to both beta (β) and gamma (γ) particle radiation, while photodiode 2 responds only to γ radiation. The subtraction of the signals from the two photodiodes yields a measure of the local energy deposition from the beta particles

Two silicon-based PIN photodiodes (S3588-08, Hamamatsu Photonics, Hamamatsu, Japan), each with a 3 × 30 mm^2^ active area, were used in the design of the radiation detector. Readout electronics were designed by Carroll and Ramsey Associates (Berkeley, CA). For our system, these diodes were operated with no externally applied bias voltage. A zero-bias voltage substantially reduces the reverse leakage or dark current that would otherwise be induced by a reverse-bias voltage. Figure 2 illustrates the geometric relationship between the two diodes. Photodiode 1 was mounted in close proximity to the tubing carrying the positron-emitting solution and generated a signal due to both positron (β) and gamma (γ) particle interactions. Photodiode 2 was mounted directly behind the first, thereby effectively shielded from positrons and thus generated a signal only due to gamma particle interactions. Photodiode 1 and its ceramic mounting provided enough thickness (1.52 mm) to inhibit beta particle interactions in photodiode 2, for most common beta emitters. Electronic subtraction of the two signals enabled a measurement of radioactivity that was largely independent of ambient gamma background level by representing the local energy deposition of the positrons only. The signal resulting from this subtraction is referred to as the beta voltage. This gamma subtraction technique assures an accurate measure of radioactivity with very little dependence on the distribution of 511-keV photons produced from positron-electron annihilation in the vicinity of the radiation detector.

Signals from both photodiodes were amplified by a current-to-voltage amplifying stage. The output of the current-to-voltage gain stage of photodiode 2 was subtracted from that of photodiode 1, and a low-pass filtering stage was applied to yield a current output. The output signal was then calibrated to translate measured voltage to radioactivity concentration.

### LabVIEW algorithm

**Fig. 3:**
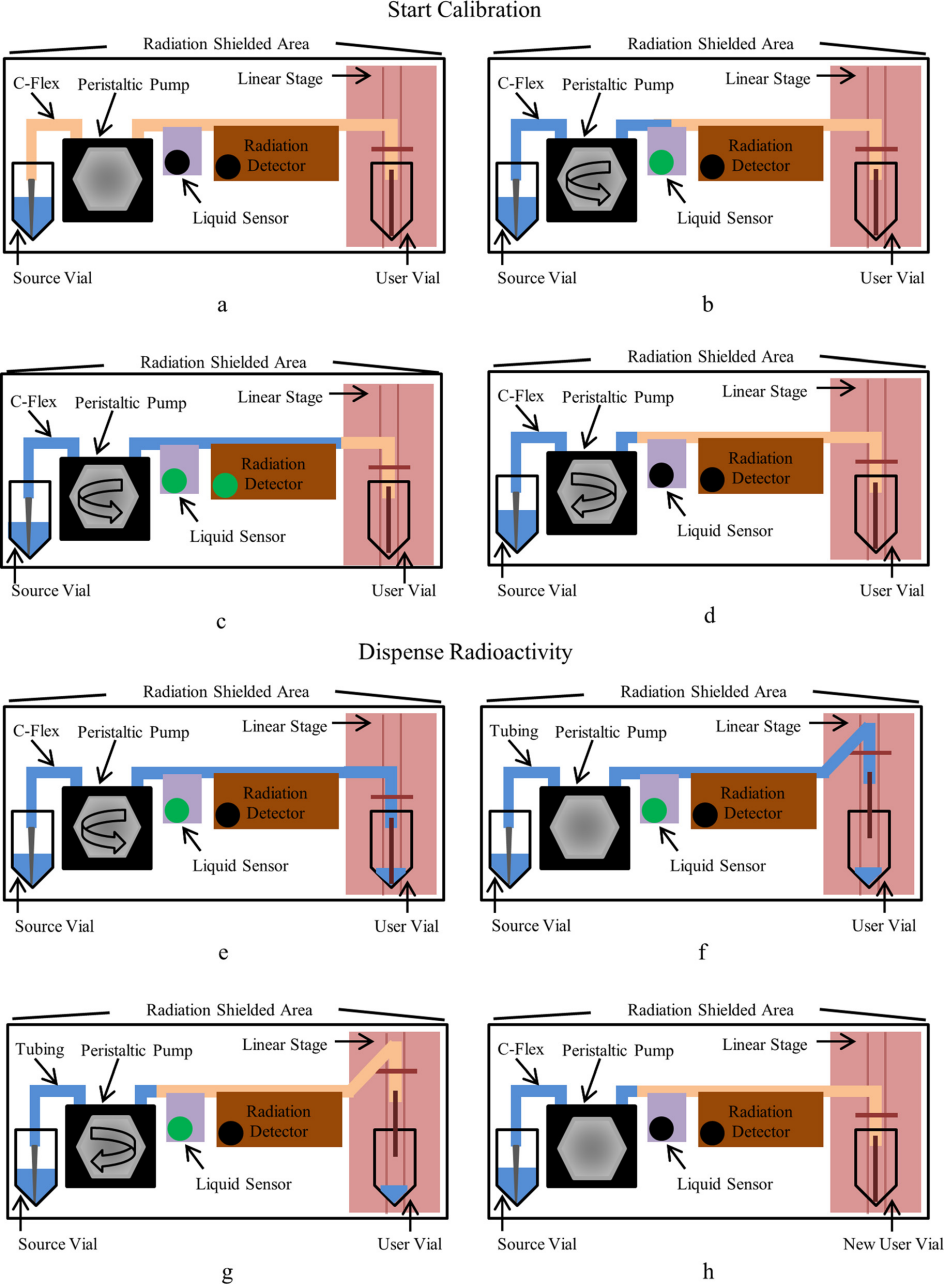
Series of diagrams showing the steps of the two routines applied to dose dispensing for radiochemistry usage. The start calibration protocol is illustrated from **a** to **d**: **a** the initial state of the dispenser, **b** the pump is actuated until the radioactivity solution reaches the liquid sensor in order to set a reference position, **c** the pump is again actuated to cover the radiation detector and the measured signal is saved to file, and **d** the pump is actuated in the reverse direction to move the radioactivity solution to a shielded region behind the reference position. The dispense radioactivity protocol is illustrated in **e**–**h**: **e** the pump is actuated to dispense the requested amount of radioactivity; **f** the stage is raised; **g** the pump is actuated in the reverse direction to reposition the radioactivity solution again to a shielded region behind the reference position; **h** after the user vial is removed from the system, a new user vial must be positioned with the stage lowered in order to dispense again

The LabVIEW algorithm was divided into two routines (1) *start calibration* and (2) *dispense radioactivity* (Fig. 3). The first procedure, start calibration, initiated the calibration process for determining a reference radioactivity concentration and reference time. This step was performed once, each time a new batch of [^18^F]fluoride solution was connected, before automated aliquoting. For radioactivity aliquoting in radiochemistry applications, this routine:

(i) Calibrated the optical sensor.
(ii) Pumped solution until the liquid sensor triggered, indicating arrival of the liquid at the sensor reference position.
(iii) Pumped a known volume of solution to completely cover the radiation detector.
(iv) Read the radiation detector and saved information to file.
(v) Retracted the radioactivity solution to a shielded region behind the reference position which served as a home position.

The second routine, dispense radioactivity, aliquoted the requested radioactivity using the calibration file created previously in the start calibration procedure and operator-specified parameters such as the requested quantity in units of volume or radioactivity. For radioactivity aliquoting in radiochemistry applications, this routine:

(i) Moved the solution from the home position to the location of the tip of the tubing based on the linear flow rate and the volume of tubing in between.
(ii) Dispensed the requested radioactivity/volume and appended data to a file for record-keeping purposes.
(iii) Raised the stage and retracted the radioactivity solution to home position.

The user must ensure that the linear stage was lowered before initiating the dispense radioactivity routine. The algorithm development for the dispenser system required independent calibration data from the radiation detector as well as the peristaltic pump. Specifically, the output voltage from the radiation detector was calibrated to known radioactivity concentration, as measured by a dose calibrator and a precision scale. Similarly, the speed control voltage from the peristaltic pump was calibrated to flow rate, as measured by a precision scale and the known density of the liquid solution. Calibration data from the peristaltic pump and radioactivity detector were incorporated into the dispensing algorithm.

### Radiation detector response

Two variations of the readout electronics for the radiation detector were considered for possible applications of ARAS. Specifically, the dispenser was assessed as a tool (1) to dispense a desired radioactivity or volume of [^18^F]fluoride in [^18^O]oxygen-enriched water ([^18^O]H_2_O) for laboratories in which each batch of radioisotope is used for multiple research projects or production runs (typical radioactivity concentration ~0.1–1 mCi μL^−1^) and (2) to infuse a prescribed dose of an [^18^F]fluoride-labeled probe through a catheterized mouse tail vein (typical radioactivity concentration ~1–10 μCi μL^−1^). For these distinct applications, the readout electronics of the radiation detector were identical except for a change in the gain of the current-to-voltage amplifier stage (see “Radiation detection technique” section). The “high”-gain and “low”-gain configurations varied in gain by a factor of ~20. A higher-gain configuration allows for an increased sensitivity. However, the higher-gain configuration is also susceptible to electronic saturation when high radioactivity concentrations are used, setting an upper limit of useable concentrations. To avoid this situation, selection of the operation range of the radiation detector must precede its use, according to predetermined application specifications. In our case, these two configurations were deemed adequate for these two extreme examples of use.

Both configurations were characterized to determine the relationship between voltage signal and radioactivity concentration. C-FLEX tubing (ID = 0.508 mm) was filled with 3 and 60 μCi μL^−1^ of an [^18^F]fluoride solution at the start of measurement for the high- and low-gain configurations, respectively, and secured over the radiation detector’s sensitive area.

The minimum detectable activity (MDA) specifies the lowest amount of radioactivity that can be reliably measured [4]. The MDA, in units of microcurie per microliter, was determined by requiring a minimum signal to noise ratio of 5 for the measured voltage. The minimum voltage was calculated as follows:

1 $$ \mathrm{minimum}\ \mathrm{voltage}={V}_{\mathrm{b}}+5{\sigma}_{\mathrm{b}} $$

where *V*_b_ is the average background voltage and *σ*_b_ is the standard deviation in the background voltage. Based on the independent calibration of voltage versus radioactivity concentration, the minimum voltage was then converted to a radioactivity concentration to yield the MDA.

### Validation of dispensed volume

To assess volume dispensing performance, volumes of water spanning between 1 and 300 μL were requested using the designed algorithm. Each volume measurement, based on the weight of the dispensed solution, was performed a total of three times. For each measurement, the percent difference, *PD*_vol_, was calculated as follows:

2 $$ P{D}_{\mathrm{vol}} = 100\kern0.5em \times \kern0.5em \frac{V_{\mathrm{d}}-{V}_{\mathrm{r}}}{V_{\mathrm{r}}} $$

where *V*_d_ is the dispensed volume and *V*_r_ is the requested volume. An Excellence Plus XP Analytical Balance (XP205, Mettler Toledo, Columbus, Ohio) was used to measure volume given the density of water of 1.00 g cm^−3^.

### Assessment of sterility of dispensed solutions

To assess sterility of the system and dispensing procedure, several samples of [^18^F]FDG were dispensed into sterile empty vials and the samples were tested via standard United States Pharmacopeia methods. Aseptic handling procedures were used during installation of the source vial and the collection vial. After a 24-h period for radioactive decay, two 100-μL aliquots were taken from each sample and mixed with soybean casein digest medium and fluid thioglycollate medium, respectively, and incubated for 14 days at 37 °C.

### Assessment of residual radioactivity after dispensing

Residual radioactivity was tested using a [^18^O]water/[^18^F]fluoride solution. After dispensing multiple samples, liquid was automatically retracted from the needles and tubing by running the pump in reverse. The tubing (including the needles) was then removed and assayed in a dose calibrator. The residual activity was compared with the starting activity (after correcting for radioactive decay). The starting radioactivity amounts were 237, 91.6, and 255 mCi, each in a volume of 1 mL.

### Test application I

ARAS was installed to provide an automated system for [^18^F]fluoride dispensing in a radiochemistry facility. To assess radioactivity aliquoting performance, [^18^F]fluoride in a solution of [^18^O]H_2_O at an initial radioactivity concentration of 60 μCi μL^−1^ was used. While this radioactivity concentration is on the lower side of what is typically produced from the cyclotron, this concentration facilitated examination and handling of the detector with small amounts of radioactivity. Dispensing of radioactivity amounts of 10, 6, 4, and 2 mCi were requested. The aforementioned radioactivity amounts corresponded to volumes greater than 20 μL in order to minimize error due to dispensing of small volumes. Each radioactivity sample was measured four times in a CRC-25PET dose calibrator (Capintec, Ramsey, NJ), with the sample repositioned between measurements. The radioactivity percent difference, *PD*_rad_, was calculated for each measurement as follows:

3 $$ P{D}_{\mathrm{r}\mathrm{ad}} = 100\kern0.5em \times \kern0.5em \frac{R_{\mathrm{d}}-{R}_{\mathrm{r}}}{R_{\mathrm{r}}} $$

where *R*_d_ is the dispensed radioactivity measured with the dose calibrator and *R*_r_ is the requested radioactivity.

### Test application II

ARAS was also evaluated for infusing a selectable amount of a PET probe into a mouse via the tail vein. [^18^F]FDG was loaded into the source vial of the dispensing system. The start calibration routine (“LabVIEW algorithm” section) was modified so that at the end of the routine, the tubing was primed with the [^18^F]FDG solution. The mouse tail vein was catheterized, and the catheterization tubing was connected to the C-Flex pump tubing, with care taken to avoid an air pocket. A total of 100 μCi of [^18^F]FDG was requested for dispensing. The dispense radioactivity routine (“LabVIEW algorithm” section) was modified to exclude linear stage motion as well as exclude retraction of the radioactivity after injection. The entire anesthetized mouse was placed in the dose calibrator, and the dispensed radioactivity was confirmed. For imaging, the mouse was then placed in a custom-designed holder [5] and scanned in an Inveon preclinical PET tomograph (Siemens Preclinical Solutions, Knoxville, TN). To properly quantify the total activity in the mouse, attenuation correction was performed. To achieve this, the mouse was scanned with an X-ray MicroCT (Siemens Preclinical Solutions, Knoxville, TN). PET emission images were reconstructed with OSEM with attenuation correction applied. The experiment was performed a total of three times on three different mice.

## Results and discussion

### Radiation detector response

**Fig. 4:**
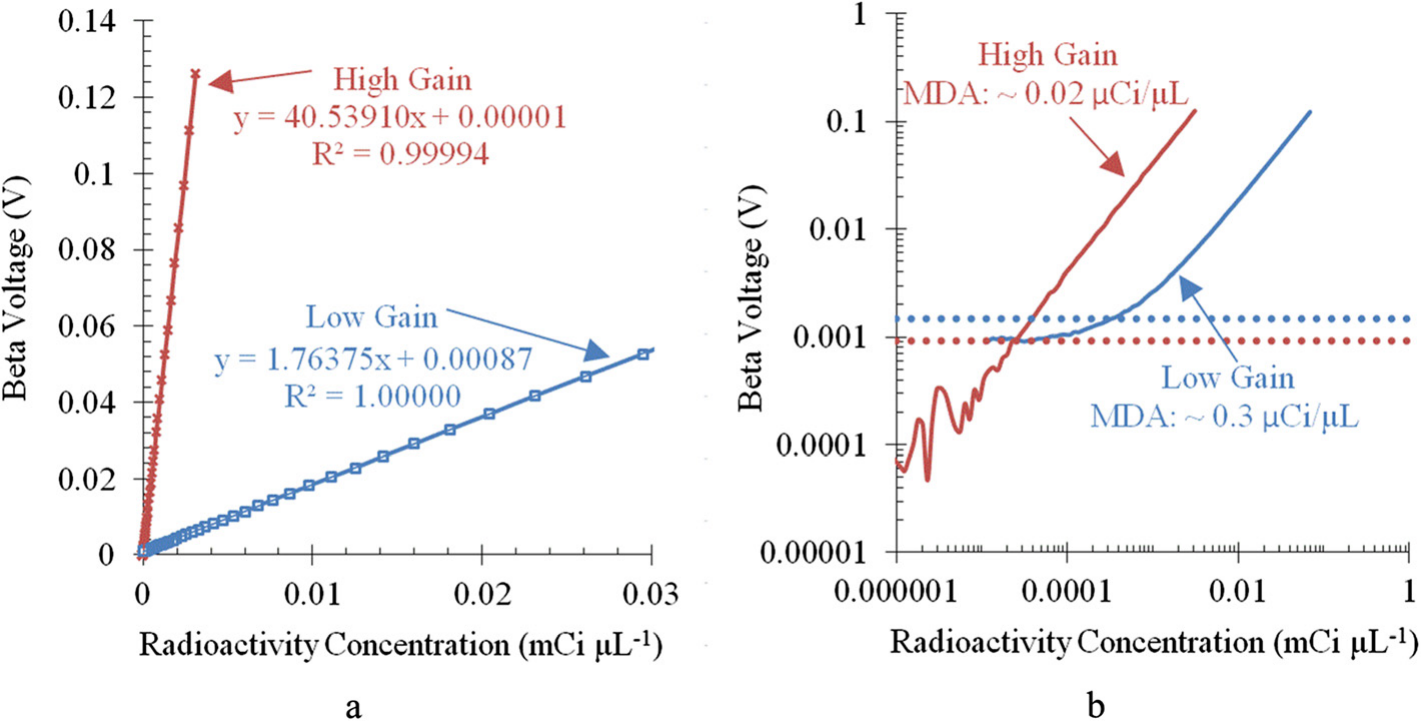
Determination of the dynamic range of ARAS. Signal calibration curves on a **a** linear scale and **b** log scale. On the log scale, *blue* and *red dotted lines* correspond to the minimum voltage used to determine the MDA for the low-gain and high-gain configurations, respectively

Figure 4a shows that a linear relationship exists between radioactivity concentration and the detector voltage signal due to beta particle interactions (henceforward referred to as the beta voltage). This linear relationship validates the use of this specific radiation detector to measure concentration. In order to properly use the radiation detector though, it is necessary to fully understand its detection limits at both the low end of radioactivity and the high end of radioactivity.

The minimum detectable activity (MDA) specifies the lowest amount of radioactivity that can be measured [4]. To better evaluate the behavior of the radiation detector at low radioactivity concentrations, Fig. 4b shows the calibration data plotted on a log scale. A visual comparison of the two curves confirms a lower MDA available with the high-gain configuration compared to the low-gain configuration. The minimum voltage was converted to a radioactivity concentration using the equations shown in Fig. 3a to yield the MDA, which was 0.02 μCi μL^−1^ for the high-gain configuration and 0.3 μCi μL^−1^ for the low-gain configuration.

The maximum detectable activity specifies the highest amount of radioactivity that can be measured reliably. The radiation detector output saturated at 5 V. Extrapolation of the calibration data to 5 V yielded a maximum detectable activity of 120 and 2830 μCi μL^−1^ for the high-gain and low-gain configurations, respectively.

A summary of the minimum and maximum detection limits for the two configurations is shown in Table 1. These values are dependent on the individual pieces of tubing used as slight variation in tubing inner and outer diameter dimensions can lead to significant changes in values. Nonetheless, in both cases, the dynamic range of the radiation detector was determined to span four orders of magnitude. The calibration step for each source vial of radioactive solution ensures that these variations do not affect the accuracy of the dispensed radioactivity, although the exact dispensed volume might be variable.

**Table 1:** Minimum and maximum detectable activities. The dynamic range of the radiation detector spans four orders of magnitude

ARAS radiation detection limits		
Gain	Minimum detectable activity (μCi μL^−1^)	Maximum detectable activity (μCi μL^−1^)
High	0.02	120
Low	0.3	2830

### Validation of dispensed volume

**Fig. 5:**
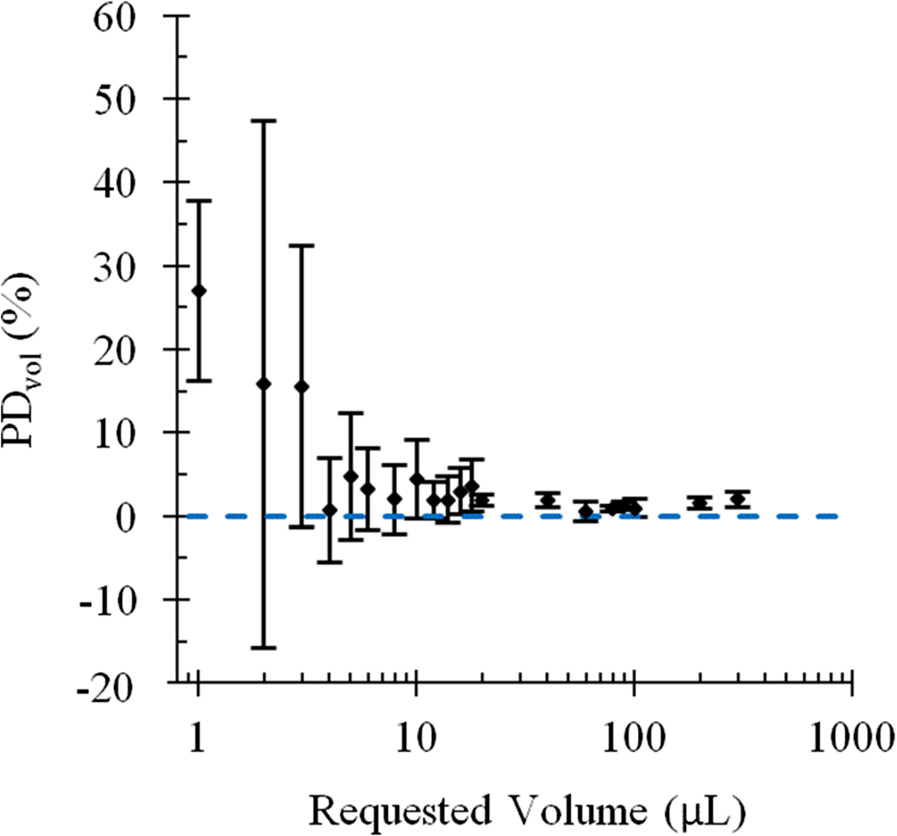
Volume percent difference (*PD*_vol_) as a function of requested volume. For volume requests greater than 20 μL, the percent error is within 5 %. The percent error increases to within 10 % at a volume of 5 μL. Below 4 μL, the dispensed volume is highly variable

Accurate and precise control of the peristaltic pump is critical in order to dispense the requested volume. This volume may be requested explicitly by the user or calculated from a requested radioactivity and the reference measurement of initial radioactivity concentration provided by the radiation detector. Figure 5 shows *PD*_vol_ over the three measurements we performed in this work. The error bars correspond to the standard deviation of *PD*_vol_ calculated from the three measurements. For volume requests greater than 20 μL, the percent error is within 5 %. The percent error increases to within 10 % at a volume of 5 μL. Below 4 μL, the dispensed volume is highly variable.

### Assessment of sample sterility

Since, in some applications, the dispensed samples would be used in small animals, or potentially even human subjects, it is critical that sterility be preserved during operation. Sterility testing was performed for three dispensed samples, and no evidence of bacterial or fungal growth was observed after the incubation period. Furthermore, the tubing is in principle disposable, providing another way that sterility can be maintained.

### Residual radioactivity after dispensing

If the dispenser is used to aliquot different source solutions (e.g., different batches of radioisotope, or different PET probes), one must consider the effect of carryover. The amount of carryover was characterized using [^18^F]fluoride solution. From measurements taken on three separate occasions, 0.20 ± 0.06 % (*n* = 3) of the initial activity (corrected for radioactive decay) remained in the tubing and needles after the dispensing process (including retraction of liquid after dispensing). This corresponds to a residual volume of 2.0 ± 0.06 μL (*n* = 3). Depending on the distribution of this residual liquid within the fluid path, it could impact the calibration process when the second source solution is loaded since the volume of the detector region is only 6.1 μL. A simple way to mitigate this problem is to replace the tubing when switching from one source solution to another.

### Test application I

**Fig. 6:**
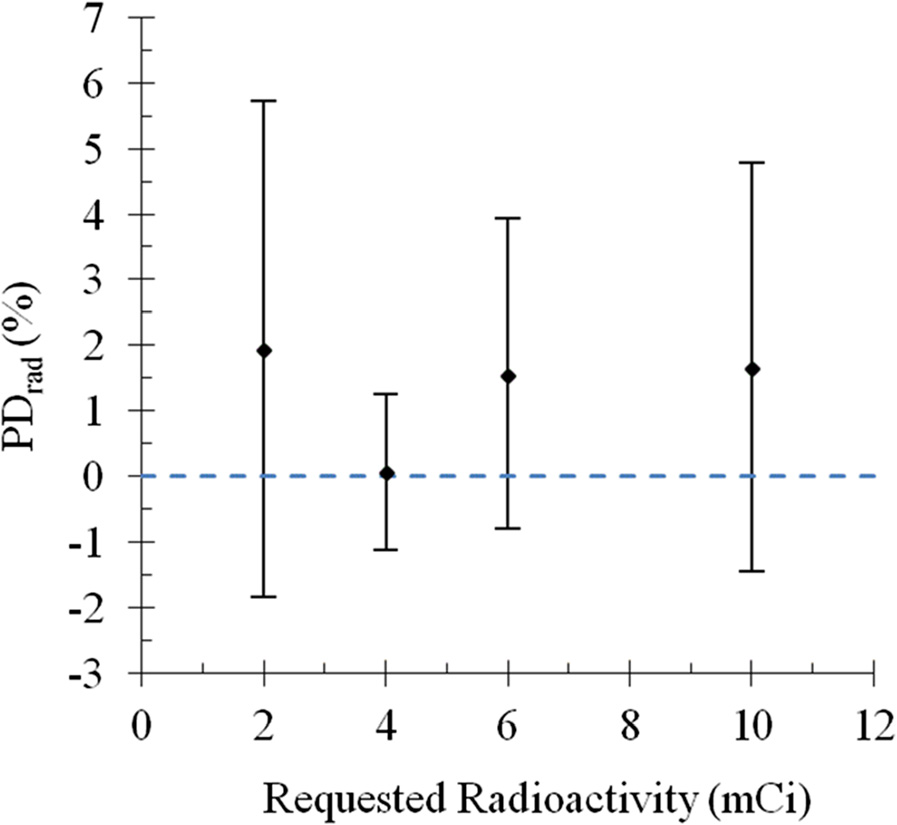
Radioactivity percent difference (*PD*_rad_) as a function of requested radioactivity. While the average *PD*_rad_ across the four measurements is within 2 % from the requested amount, variations around this mean can be up to ±4 %

At the Crump Institute for Molecular Imaging, multiple radiochemists draw [^18^F]fluoride in [^18^O]H_2_O from a source vial obtained daily from the cyclotron. Conventionally, the staff (1) manually draws [^18^F]fluoride using a syringe, (2) assays the manually drawn amount using a dose calibrator, and (3) iterates between drawing and assaying [^18^F]fluoride until the desired amount is collected. Even if a radiochemist needs only a small amount of radioactivity, he/she is exposed to radiation due to the total amount of radioactivity in the source vial. As the dispenser automatically draws and assays the radioactivity, the user exposure to radiation is reduced. Furthermore, the accuracy of dispensing is increased, the variability between sequential draws is reduced, and record keeping becomes automated. Figure 6 shows the *PD*_rad_ averaged over the four measurements for each sample. Error bars correspond to the standard deviation of *PD*_rad_ calculated from the four measurements. While the average *PD*_rad_ across the four measurements is within 2 % from the requested amount, variations around this mean can be up to ±4 %. Given the intrinsic variability of dose calibrators [6, 7], we consider this as a good result.

### Test application II

**Fig. 7:**
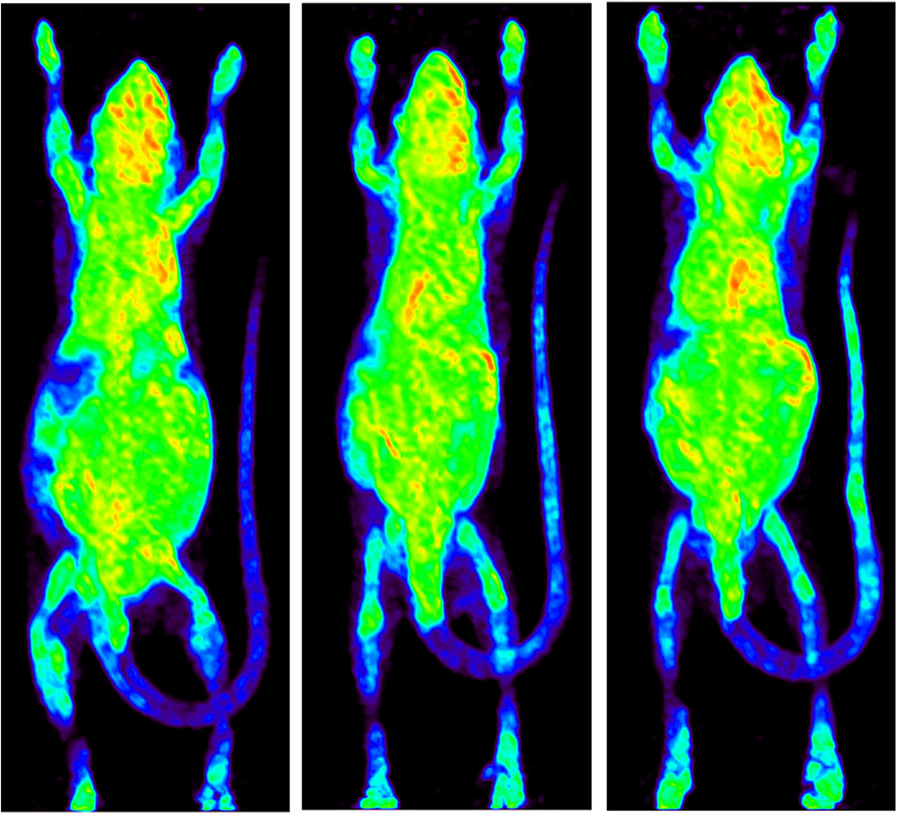
PET image produced from an [^18^F]FDG infusion using the automated dispenser. Images are saturated in order to view the tail. Region of interest analysis on the attenuation corrected images show that the percent of radioactivity in the tail is no more than 2 % of the total radioactivity injected, indicating a good infusion

Another potential application for ARAS is to interface it with an automated vascular access system for mouse tail vein injections of PET probes. Such a system is currently being developed in our group [8]. The dispenser was evaluated for use with this system, via three mouse tail vein injections performed in a single day. The dispenser was programmed to deliver 100 μCi into the catheter that was already inserted in a mouse tail vein. After injection, the three mice were individually placed in a dose calibrator which measured an average of 102.5 ± 0.5 μCi decay corrected, in a reasonable agreement with the requested amount. PET imaging data were subsequently acquired, after a 1-h uptake period, and Fig. 7 shows the resulting [^18^F]FDG PET images. The region of interest analysis on the attenuation-corrected images showed that the percent of radioactivity in the tail was no more than 2 % of the total radioactivity injected, indicating a good infusion.

## Conclusions

We developed ARAS, an automated radioactivity aliquoting system for dispensing solutions containing positron-emitting radioisotopes with particular focus on fluorine-18 (^18^F). The key to the operation of this system was a solid-state detector integrated in line with a peristaltic pump and computerized control of the motion of liquids in the calibrated system. The system demonstrated volume accuracy within 5 % for volumes of 20 μL or greater. When considering volumes of 20 μL or greater, delivered radioactivity was in good agreement with the requested radioactivity as measured independently with the dose calibrator. The detector operates in a DC current mode, where the radiation-induced photo-current is simply averaged over time to produce a steady signal proportional to the average rate of energy deposited in the Si diode. Thus, the response is insensitive to changes in normal lab temperatures where extreme changes in temperature are not expected. Moreover, the dual-diode scheme provides a measure of self-correction, since both front and rear diode channels are subject to the same changes in temperature.

The integration of the detector and pump led to a flexible system that can accurately dispense solutions containing radiolabeled probes in radioactivity concentrations directly produced from a cyclotron (~0.1–1 mCi μL^−1^), to lower activity concentrations intended for preclinical mouse scans (~1–10 μCi μL^−1^). Such a system has the potential to significantly reduce the exposures of personnel handling radioactive solutions for biomedical research or clinical applications, while at the same time streamline the workflow. Its small size and low cost offer an opportunity for multiple copies of such a system to be installed at the many steps along experiments utilizing radioactive solutions, where manual operations currently take place. The implementation of ARAS within a protocol that ensures sterility of the disposable tubing demonstrates a promising approach for radiation handling in application related to PET involving patients or animal studies.
